# Prevalence study of intermittent hormonal therapy of Prostate Cancer patients in Spain

**DOI:** 10.12688/f1000research.53875.2

**Published:** 2022-04-21

**Authors:** Xavier Bonfill-Cosp, Ariadna Auladell-Rispau, Ignasi Gich, Javier Zamora, Luis Carlos Saiz, Jose Ignacio Pijoan, Iratxe Urreta, José Antonio Cordero

**Affiliations:** 1 Iberoamerican Cochrane Centre, Biomedical Research Institute Sant Pau (IIB Sant Pau), Barcelona, Spain; 2 CIBER of Epidemiology and Public Health (CIBERESP), Madrid, Spain; 3 Autonomous University of Barcelona, Barcelona, Spain; 4 Clinical Biostatistics Unit, Hospital Universitario Ramón y Cajal, Madrid, Spain; 5 Institute of Metabolism and System Research, University of Birmingham, Birmingham, UK; 6 Unit of Innovation and Organization, Navarre Health Service, Pamplona, Spain; 7 Clinical Epidemiology Unit, Hospital Universitario Cruces/BioCruces-Bizkaia Health Research Institute, Barakaldo, Spain; 8 Clinical Epidemiology and Research Unit, University Hospital of Donostia, Donostia, Spain; 9 School of Health Sciences Blanquerna, University Ramon Llull, Barcelona, Spain

**Keywords:** intermittent androgen deprivation therapy (IAD), LHRH analogues, prostate cancer, appropriateness

## Abstract

**Background: **Although intermittent androgen deprivation therapy was introduced many years ago to improve patients’ quality of life with the same carcinologic efficiency as continuous hormonal therapy, recent data suggest that intermittency could be underutilised. This study aims to estimate the prevalence of prostate cancer patients receiving intermittent androgen deprivation therapy in Spain.

**Methods: **A retrospective, longitudinal study was conducted using electronic drug dispensation data from four Spanish autonomous communities, which encompass 17.23 million inhabitants (36.22% of the total population in Spain). We estimated intermittent androgen therapy use (%IAD) and the prevalence of patients under intermittent androgen therapy in reference to the total number of PC patients using hormonal therapy (P _IAD_) and stratified by region. Other outcome variables included the pharmaceutical forms dispensed and the total direct annual expenditure on androgen deprivation therapy‐associated medications.

**Results: **A total of 863,005 dispensations corresponding to a total of 65,752 men were identified, treated with either luteinizing hormone-releasing hormone (LHRH) analogues (353,162) administered alone or in combination with anti‐androgens (509,843). Overall, the mean (±SD) age of the patients was 76.9 (±10.4) years. Results revealed that the mean annual P _IAD_ along the study was 6.6% in the total population studied, and the overall %IAD during the five‐year study period was 5.6%. The mean cost of hormonal therapy per year was 25 million euros for LHRH analogues and 6.3 million euros for anti-androgens.

**Conclusions: ** Few prostate cancer patients in Spain use the intermittent androgen deprivation therapy suggesting underutilization of a perfectly valid option for a significant proportion of patients, missing the opportunity to improve their quality of life and to reduce costs for the National Health Service with comparable overall survival rates than continuous therapy.

## Abbreviations

ADT: Androgen Deprivation Therapy

ATC: Anatomic Therapeutic Code

CAD: Continuous Androgen Deprivation therapy

ELD: Effect duration of the Last Dose

GPC: Clinical Practice Guideline

IAD: Intermittent Androgen Deprivation therapy

%IAD: Percentage of time off treatment

IntT: Interval Time

LHRH analogue: Luteinizing Hormone-Releasing Hormone analogue

PCa: Prostate Cancer

P _IAD_: Prevalence of Intermittent Androgen Deprivation therapy

PIIT: Potentially Intermittent Interval Time

PSA: Prostate Specific Antigen

## Introduction

Androgen deprivation therapy (ADT) is the main indication for Prostate Cancer (PCa) patients with metastatic disease and the most used therapeutic approach to treat patients who experience a biochemical relapse after radical prostatectomy. Reduction of circulating levels of androgen hormones can be achieved either with drugs, such as luteinizing hormone-releasing hormone analogues (LHRH) or with orchiectomy, which is irreversible. Nevertheless, ADT is associated with a wide array of adverse effects ^1^ ranging from the well-known hot flushes, loss of libido, gynecomastia, or erectile dysfunction up to midterm consequences such as bone fractures, depression, diabetes, and hematologic ^2^ and cardiovascular events. ^3^^,^^4^ Importantly, all of these undesirable effects increase with the duration of ADT. ^5^

In the 1990s, as a response to minimise these negative outcomes, intermittent androgen deprivation therapy (IAD) emerged as an alternative to continuous androgen deprivation therapy (CAD) but not without controversy regarding its feasibility. ^6^ Over the years, it has been shown that IAD can improve patients’ quality of life by reducing toxicity, ^7^ reduce costs ^8^ and potentially delay the onset of resistance to chemical castration. ^9^ Recent strong evidence from randomized clinical trials ^10^^–^^14^ and systematic reviews, ^15^ as well as favourable recommendations included in recently updated clinical guidelines, ^16^^–^^18^ highlight the potential benefits of IAD, supporting its use as an alternative treatment.

Despite the potential advantages of IAD, in a previous study we detected a low use of this approach in Catalonia (Spain) ^19^ compared to recent evidence of IAD utilization in Canada. ^20^ We conducted the current study with the same methodology but covering a much bigger area of Spain (17 million inhabitants from four autonomous communities) to assess the prevalence of IAD with real-world data.

## Methods

### Study design and setting

An observational, longitudinal study was conducted using electronic drug dispensation data from four Spanish autonomous communities: Catalonia, Madrid, Basque Country and Navarra (population: 16.05%, 14.17%, 4.63%, and 1.37% of the total population in Spain respectively, representing overall 36.22% of the total population in Spain, which was about 47 million inhabitants in 2020).

### Data sources

Data used for this study were obtained from the health government agencies from each of the four territories in Spain: CatSalut in Catalonia, Servicio Madrileño de Salud in Madrid, Osakidetza in the Basque Country and Servicio Navarro de Salud-Osasunbidea in Navarra. We received all relevant dispensation data from the four respective agencies for the period between 1 January 2011 and 31 December 2016. Databases contained anonymised patient codes, date of birth, dispensing date, prescriber code (anonymised), drug name and anatomic therapeutic code (ATC), total dose per pharmaceutical form, number of dispensed drug units and the corresponding cost. The dispensation of each of the following LHRH analogues was identified: leuprorelin (L02AE02), triptorelin (L02AE04), goserelin (L02AE03), buserelin (L02AE0), histrelin (H01CA0), nafarelin (H01CA02), and the following antiandrogen drugs: bicalutamide (L02BB03) and flutamide (L02BB0), in any pharmaceutical form or dose registered in Spain. We selected ≥ 18-year-old male patients with at least one dispensation of LHRH analogues. We excluded individuals under age 18 based on the high likelihood that LHRH dispensations in this subgroup would be associated with precocious puberty, which was not within our interest. Given the specificity of ADT, we assumed that all other men ≥ age 18 were prescribed these drugs as PCa treatment.

The requested data were provided under the requirements of the Spanish data protection laws and no personal data was available to the investigators.

### Exposure parameters

**Figure 1.**
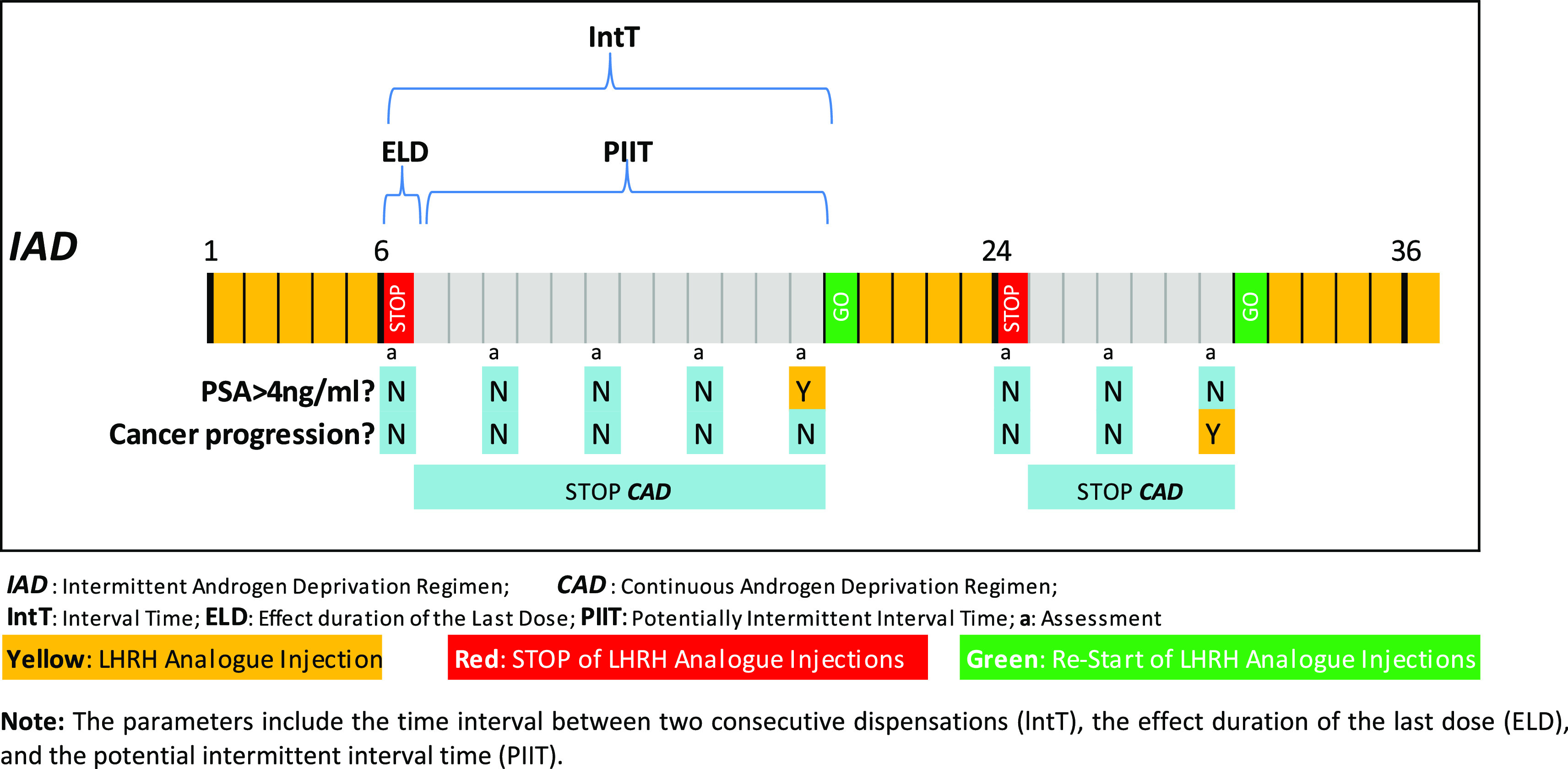
Representation of the parameters needed to identify the intermittent periods in a 36-month scenario.

To distinguish between the CAD and IAD regimens, we identified all the periods during which patients received no LHRH analogue or Potentially Intermittent Interval Times (PIIT). We first identified all interval times (IntT), defined as the period between any two consecutive dispensations for each patient, together with the effect of the last dose (ELD), identified as the time period during which the patient was covered by the medication, as shown in Figure 1. Further details about the methods followed can be consulted in our previous publication. ^19^ Table 1 summarizes the type of LHRH analogue used, the total dose of the commercially available formulation, and the corresponding total duration of action of each dose. Although an intermittency gap usually starts after a minimum of six months of continuous hormonal treatment to reconfirm patient castration (PSA < 4 ng/mL), in our study, we considered three months as the minimum potential gap in order to have a conservative approach in IAD estimation.

**Table 1.** LHRH analogue sustained-release formulations used, including dose and expected duration of action.

	Dose, mg												
	≤2	3.30	3.60	3.75	6.60	7.50	9.90	10.80	11.25	22.5	30	45	50
Busereline	1 d *	1 m *	-	-	2 m	-	3 m	-	-	-	-	-	-
Gosereline	-	-	-	-	-	-	-	3 m	-	-	-	-	-
Histreline	-	-	1 m	-	-	-	-	-	-	-	-	-	12 m
Leuproreline	1 d	-	-	1 m	-	2 m	-	-	3 m	6 m	6 m	6 m	-
Triptorelin	1 d	-	-	1 m	-	-	-	-	3 m	6 m	-	-	-
Nafarelin	1 d	-	-	-	-	-	-	-	-	-	-	-	-

* Exposition measure: d = day/s, m = month/s.

### Data analyses

One of the two key study variables created to estimate the use of intermittence was the percentage of time off treatment (%IAD). This percentage was calculated by dividing the sum of all the off periods (in patients with ≥1 intermittent interval time) by the sum of all interval times on any LHRH analogue regimen (i.e., the sum of periods between the first and the last dispensation dates for each patient in the study). The %IAD was calculated overall and for each region along the study period. The other key variable to estimate the number of patients under intermittency was the prevalence of IAD (P _IAD_), calculated as the number of patients with ≥1 PIIT per year, divided by the total number of patients treated with LHRH analogues in the same year. Nevertheless, it should be noted that the P _IAD_ for the first year of data (2011) was underestimated due to the prior three-month intermittency requirement (e.g., patients in the off period in January 2011 could not be identified as intermittent until the next off period). Because of that, the data from 2011 was not included in the specific analysis of intermittency. We present the costs of hormonal treatment, including LHRH analogues and anti-androgenic therapeutic groups, per drug, year, and autonomous community. We excluded the Basque Country for the calculation of the annual expenditure because of its lack of complete 2011 data. To compare the cost between communities, we normalised the data as euros per dispensation. We expressed baseline characteristics, as mean ± standard deviation for continuous variables and frequency (%) for categorical variables with the corresponding 95% confidence interval (95%CI). A chi-square test was used for comparing presence or absence of intermittency (dichotomous) depending on the four communities or the five years of follow-up (2012-2016). P values <0.05 were considered statistically significant. Calculations were performed using SPSS version 26.0 software (IBM, Armonk, NY).

## Results

**Figure 2.**
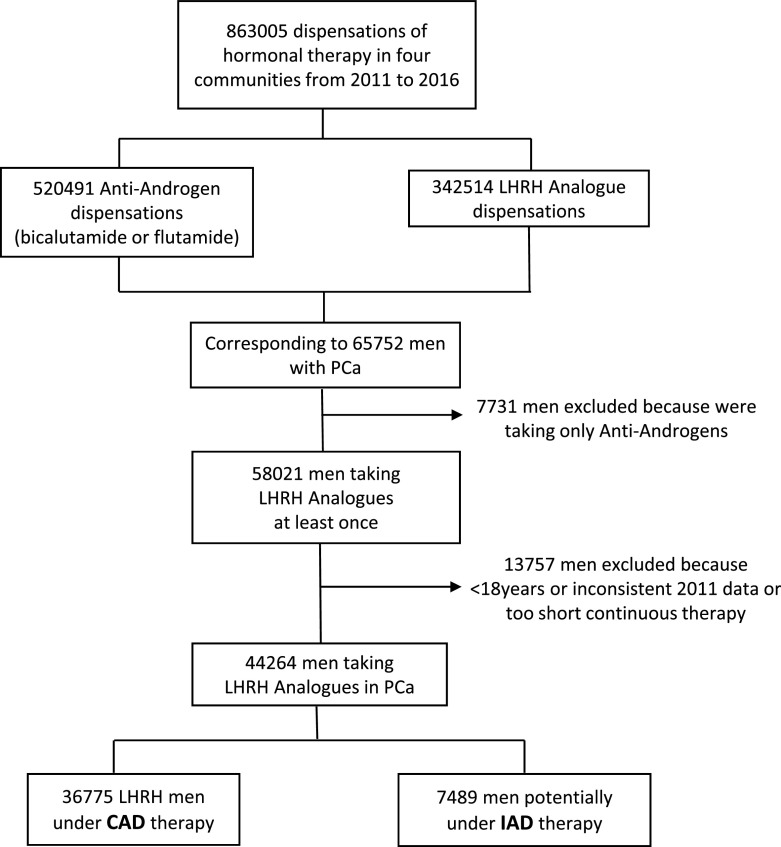
Patient distribution flowchart.

For the full study period (2011-2016), a total of 863,005 dispensations corresponding to a total of 65,752 men, including either LHRH analogues administered alone (353,162) or in combination with anti-androgens (509,843), were made in Catalonia, Madrid, Basque Country and Navarra. Previously, 309 males under the age 18 were excluded together with 7,731 subjects that were dispensed only with antiandrogens, because complete castration was not guaranteed. Additionally, 2012 data from the Basque Country were considered inconsistent (likely incomplete electronic system introduction in this area) and were also excluded from the analysis. Finally, a total of 44,264 PCa patients were included in the study, as summarized in Figure 2. Overall, the mean (±SD) age of the patients was 76.9 (±10.4) years, although 81% were older than 70 years and 47% older than 80 years.

**Figure 3.**
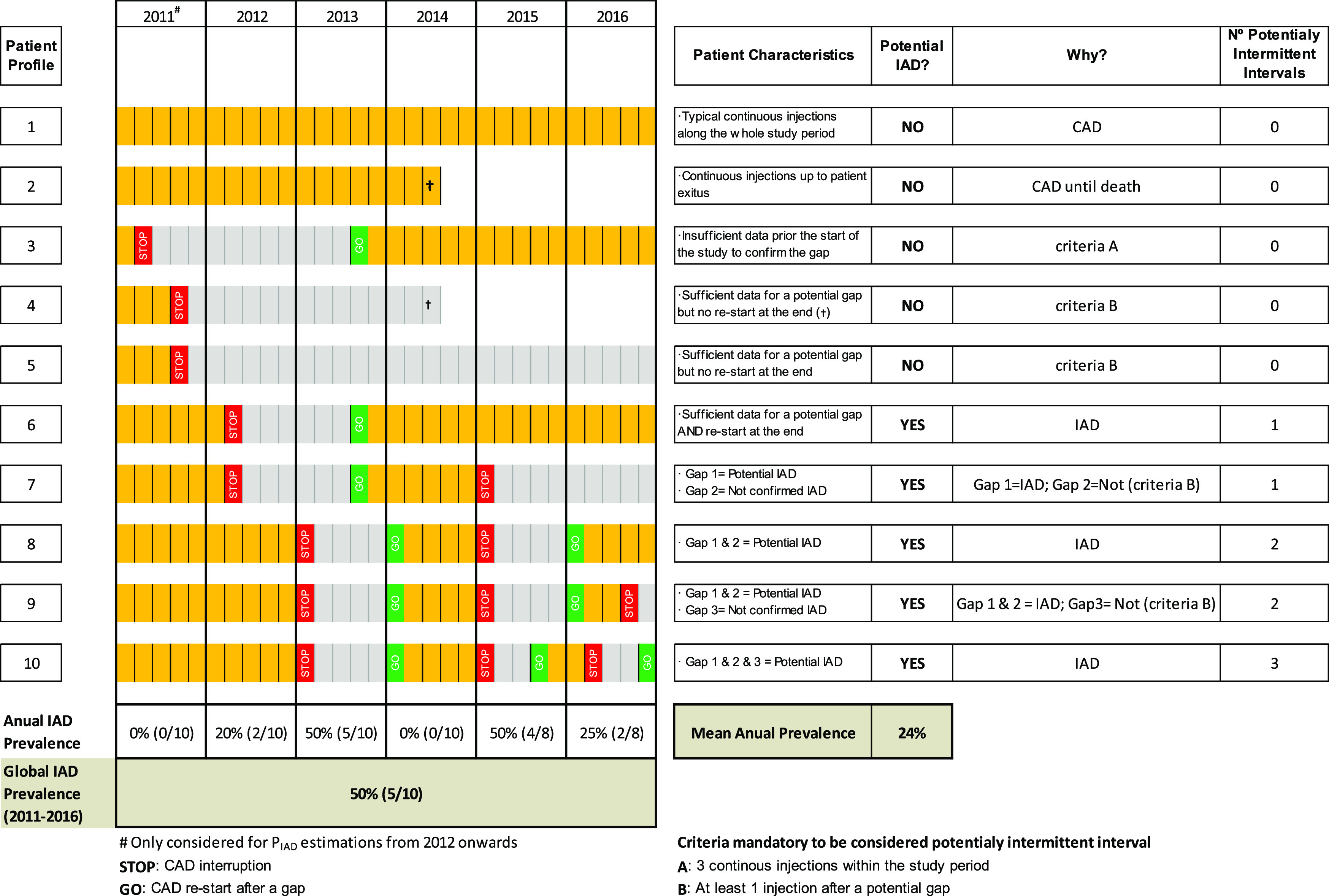
Simulation of 10 putative subject profiles in terms of CAD or IAD.

**Figure 4.**
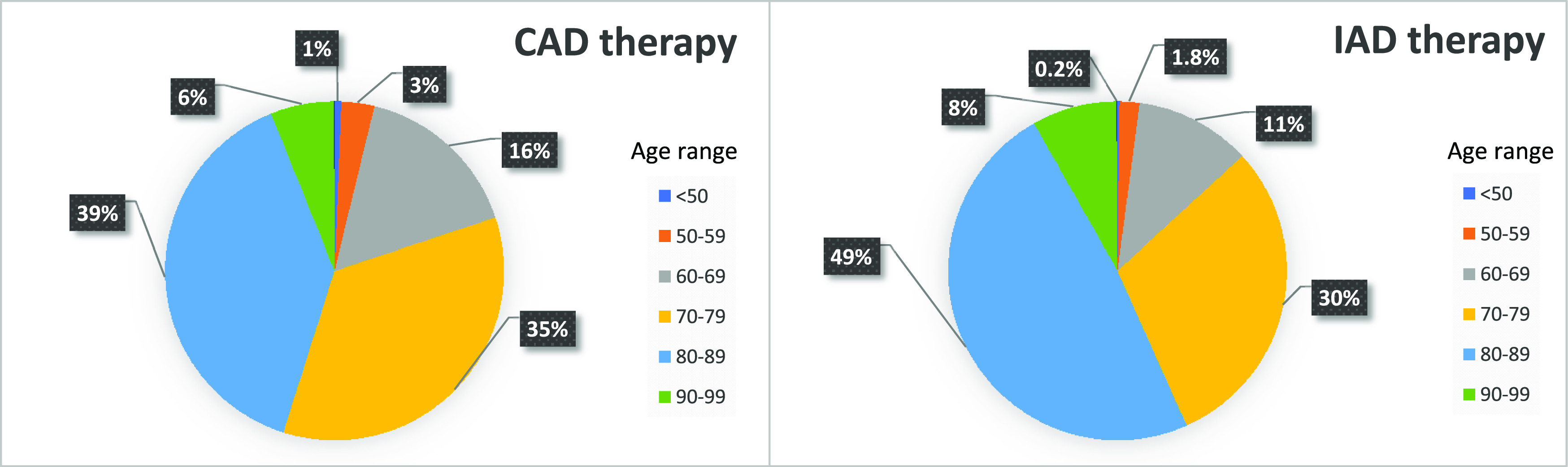
Age distribution of prostate cancer patients under IAD or under CAD.

Of the total men (44,264) finally included in the study and taking LHRH analogues alone, 36,775 (83%) were under CAD therapy and 7,489 (17%) under IAD therapy, considering the full five-year period of the study. However, this value of global prevalence represents an overestimation of the actual annual prevalence of IAD, as can be observed in Figure 3. For example, among the ten patient profiles simulated, only five profiles (6 to 10) fulfilled IAD requirements at least once (5/10 cases where “Potential IAD = Yes”), when considering the six years globally. Then, the global IAD prevalence was of 50%. However, when considering each year separately, the annual prevalence varied from 0 to 50%, with a mean annual prevalence of 24%. The age distribution of the patients according to the treatment regimen (IAD vs CAD) is shown in Figure 4. Patients on the IAD regimen were somewhat older compared to the total patients under continuous hormonal therapy. The proportion of octogenarians and older patients under IAD therapy (56.7%) was higher compared to those under CAD (45.1%).

The five-year prevalence (P _IAD_) of patients under an IAD regimen was 6.6% in the total population studied. The global prevalence of intermittency slightly increased from 8.2% in 2012 to 8.4% in 2013, but later decreased to 3.6% in 2016. We also calculated the %IAD during the five-year study period with a total %IAD value of 5.6%, which is rather consistent with the P _IAD_ figure reported earlier.

Statistically significant differences (P < 0.0001) were observed in prevalence of intermittency among the four Spanish regions and among the five years of study (2012-2016). The P _IAD_ shows that in 2012 Madrid had the highest prevalence of IAD therapy (10.7%), followed by Navarra (8.2%) and Catalonia (5.8%). Peak values obtained were up to 14.2% [2014] in Madrid, 8.5% [2013] in Navarra, 8.4% [2013] in Catalonia, and 3.8% [2015] in the Basque Country; thereafter, the use of intermittent treatment decreased to 4.3% by 2016 in Madrid, 4.0% in Catalonia, 3.4% in Navarra and 2.5% in the Basque Country. Table 2 shows the total P _IAD_ distributed per community per year, as well as %IAD for each community.

**Table 2.** Estimations of hormonal treatment prevalence.

Autonomous community	N° PCa under hormonal treatment	Hormonal treatment prevalence # (%)	%IAD	P _IAD_					
				2012	2013	2014	2015	2016	Total
Catalonia	33650	1.35	5.2 (5.2-5.2)	5.8% (5.4-6.2)	8.4% (7.8-8.9)	6.6% (6.1-7.0)	6.1% (5.7-6.6)	4.0% (3.6-4.3)	**6.2%** * (6.1-6-4)
Madrid	21718	1.02	11.4 (11.3-11.4)	10.7% (10.0-11.3)	8.3% (7.8-8.9)	14.2% (13.4-14.9)	6.7% (6.2-7.3)	4.3% (3.9-4.8)	**8.9%** * (8.6-9.2)
Basque Country	7095	0.88	1.7 (1.7-1.7)	-	0.3% (0.1-0.8)	1.3% (1.0-1.8)	3.8% (3.2-4.5)	2.5% (2.0-3.0)	**2.4%** * (2.2-2.7)
Navarra	3289	1.44	4.3 (4.3-4.3)	8.2% (6.7-9.8)	8.5% (7.0-10.2)	6.8% (5.5-8.3)	4.9% (3.8-6.2)	3.4% (2.5-4.5)	**6.4%** * (5.8-7.0)
Global	**65752**	**1.18**	**5.6**	8.2	8.4	7.2	5.4	3.6	**6.6** *

# In reference to male inhabitants older than 20 years of each Autonomous Community in 2016.

* P < 0.0001 (chi-square test).

**Note:** IAD prevalence (CI 95%) based on annual PIAD and %IAD.

Abbreviations: **IAD** Intermittent Androgen Deprivation therapy; **P**_**IAD**_ Prevalence of Intermittent Androgen Deprivation therapy; **%IAD** percentage of time off treatment.

We found that the most representative non-treatment period lasted for a median of six months (ranging from 3 to 58 months) considering all four areas. In Madrid and Catalonia, the median of the PIIT intervals was 6.2 months with an IQR of 7.1 and 9.2 months respectively, while in the Navarra and Basque Country the PIIT intervals were shorter and less variable: 5.1 months (IQR 4 months) and 4.0 months (IQR 3.1 months), respectively. In the four areas the global tendency was to increase the duration of the “off” period from four (2012) up to six months (2016).

The most prescribed drug in all four communities during the five-year study period was leuprorelin, followed by triptorelin and goserelin (representing globally a 51.72%, 34.72%, and 12.33%, respectively) of the total LHRH analogues dispensed.

**Figure 5.**
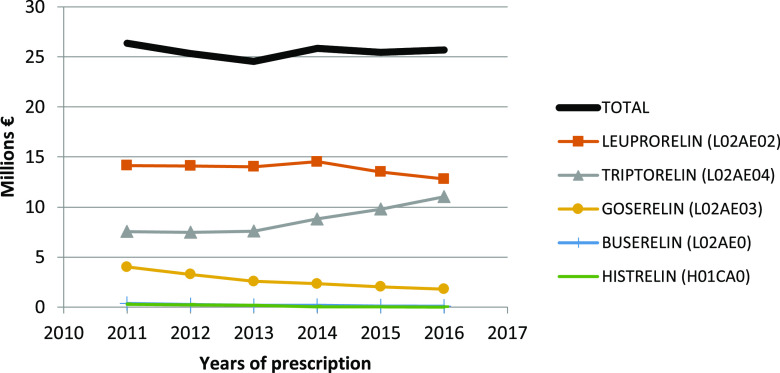
Total cost of LHRH analogues per year from 2011 to 2016 in Spain.

The global mean cost of hormonal therapy per year was 25 million euros for LHRH analogues and 6.3 million euros for anti-androgens for the four regions. Along the study period, the total expenditure in Catalonia, Madrid and Navarra decreased from 34.7 million euros [2011] down to 25.3 million euros [2016]. Figure 5 plots the changes in total cost for each specific LHRH analogue per year. The global mean (±SD) cost per dispensation was 214.6 ± 33.5 euros in 2011, then decreased to 201.7 ± 31.9 euros in 2014, and increased again up to 213.9 ± 26.3 euros in 2016.

## Discussion

Our results show that, taking either the mean P _IAD_ (6.6%) or the %IAD (5.6%) as reference, IAD use was in the range of 6% in Spain. These results are in agreement with our previous estimations from Catalonia (2008-2012): P _IAD_ = 4.2% and %IAD = 1.7% ^19^ and confirm a low global utilisation of IAD in Spain. Even worse, the IAD utilisation rate has evolved in a progressively decreasing manner along the current study period, ranging from 8.4% (2013) to 3.6% (2016). Among the four Spanish regions, the %IAD showed that Madrid had the highest prevalence of intermittency (11.4%), followed by Catalonia (5.2%), Navarra (4.3%) and the Basque Country (1.7%). The P _IAD_ showed a decreasing trend along the study period until 2016 in Madrid (4.3%), Catalonia (4.0%), Navarra (3.4%) and the Basque Country (2.5%). Based on the obtained data, we interpret that the well-known role of intermittent treatment in hormonal therapy, based on sound evidence from randomised controlled trails ^10^^–^^14^ and systematic reviews, ^15^^,^^16^ does not appear to be influencing actual clinical practice in a significant proportion of cases. It seems that the necessary trade-off between benefits and adverse effects could be much improved in those situations where IAD is as effective as CAD, since adverse effects of IAD should be lower for a broad range of morbidities. ^4^^,^^13^^,^^15^^,^^21^^–^^23^ An international survey conducted in 19 countries asking 441 physicians currently treating PCa patients, showed that 23% of non-metastatic patients treated with gonadotropin-releasing hormone analogues were using IAD. Additionally, the same authors agree with our results that the CAD and IAD use in Spain among men with non-metastatic prostate cancer, was of 35.7% and 6.9%, respectively. ^24^ Reference countries like France, Italy or Germany showed higher IAD use than Spain (9.1, 13.9 and 14.3%, respectively), still based on physicians opinions. This fact could be explained by the relatively higher healthcare pressure of physicians in Spain than in France, Italy or Germany (3.8 vs. >4.1 per 1000 people in 2015, respectively). Further evidence suggesting that the use of IAD is low in Spain, comes from data collected in Manitoba (Canada), where 74% (447/601 nonmetastatic hormone-sensitive PCa patients) were using IAD for the management of their relapse *.* ^20^

When analysing the data related to the duration of the “off” periods, or halted medication periods, we observed that the median duration varied between 4 and 6,2 months in all four regions (ranging from 3 to 13 months). These *off*-treatment periods are concordant with the corresponding values reported by Crook *et al.*, that ranged from approximately 20 months in the first non-treatment interval, down to three months in the 6 ^th^ interval. ^13^ Globally, there was also a tendency to increase the median duration of the pause period between 2012 (three to four months) and 2016 (five to six months). This may suggest that prescribers using IAD are progressively more confident with the risk/benefit balance of this therapeutic strategy. This is in agreement with the fact that patient-reported outcomes were significantly better in IAD versus CAD at 20 and 38 months of treatment, based on validated quality of life questionnaires in Japan. ^25^

We calculated that the total cost of hormonal therapy in the four autonomous communities was 31.3 million euros per year (25 million corresponding to LHRH analogues and 6.3 to anti-androgens), which represents a mean of 214 euros per dispensation. The fact that the mean cost per dispensation declined from 214.6 euros in 2011 to 201.7 euros in 2014 could be related to the fact that in 2012, the Spanish health authorities introduced restrictive measures for the sustainability of the National Health System (Royal Decree 16/2012) ^26^ and all kind medication were reduced in the whole country by as much as 12 million per month. ^27^ However, thereafter the cost increased again up to 213.9 euros per dispensation in 2016. Obviously, the efforts to enhance the use of IAD would produce important cost savings due to the reduction in expensive drug consumption, which some authors estimate in the range of one-third of CAD. ^12^ In the population studied, we would be talking of around half a million savings per million inhabitants per year.

If PCa patients are to be actively involved in their decision process related to hormonal therapy medications, as current clinical guidelines suggest, they should know the existing sound evidence that shows similar overall survival of both CAD and IAD users, but the broader span of morbidities associated to CAD. From the physicians’ point of view, the PSA levels (54%), patient request (48%), desire to maintain sexual function (40%), patient age and comorbidities (38%) were reported as the most frequent reasons for IAD use. ^24^

The main strength of our study is the capacity to analyse all public health systems electronic dispensations data from the selected Spanish regions, which eliminates any potential selection bias. They constitute a very large sample size, which favours the representativeness of the whole Spanish population. Also, the reliability of the database reduces information bias and missing data problems as previously observed by other authors. ^28^

Due to the administrative nature of our data sources, our study is not able to estimate whether patients identified under IAD are clinically appropriate or not. However, taking into account the conservative approach that we used and compared to the real-world utilization in Canada ^20^ and 19 other countries, ^24^ we could say that the use of IAD in Spain could be significantly increased, leading to the consequent improvement in patients’ quality of life ^25^ and significant savings for the National Health Service. ^10^ We plan to conduct an audit in a sample of the electronic records of PCa patients who were potential IAD candidates, to assess if they fulfilled the clinical criteria for having eventually stopped the hormonal therapy.

## Conclusions

We conclude that the IAD use in Spain was relatively low during the study period, although enough time has passed since the related international recommendations were published. As a consequence, an important proportion of hormone-sensitive prostate cancer patients could be currently missing the chance to reduce avoidable adverse effects and to improve their quality of life, apart from the important repercussion that this expense has on the health budget in times of crisis. The population-based method to estimate the IAD prevalence seems to be a quite consistent approach and opens the door to comparable international data to set up reference standards on adequate IAD utilisation.

## Data availability

### Underlying data

Project Datasphere: Intermittent hormonal therapy of prostate cancer patients in Spain: a prevalence study, https://doi.org/10.34949/fzzk-ye57. This project contains the following underlying data:

- CEIC_PROTOCOLO_Ca prostat.pdf (Study protocol)
- IAD_CRF_v1.docx
- DataDescription_v1.xlsx
- BBDD_Spain_IAD_v4.xlsx (Dispensation data)

Data are available under the terms of the PDS Data Sharing Agreement. Any individuals who wish to access the data will need to register for an account on Project Datasphere.
