# Supplementary material for: Prevalence study of intermittent hormonal therapy of Prostate Cancer patients in Spain
Source: F1000Res. 2022 Apr 21;10:1069. Originally published 2021 Oct 21. [Version 2] doi: 10.12688/f1000research.53875.2 (PMC9614286; doi:10.12688/f1000research.53875.2)
Supplement: Peer review history

# Peer review: Reviewer response for version 2

**DOI:** 10.5256/f1000research.131327.r135442

**Published:** 2022-10-27

**Authors:** Philipp Dahm

**Affiliations:** 1 Department of Urology, Minneapolis VAMC, University of Minnesota, Minneapolis, MN, USA

The authors have appropriately revised the manuscript; I have no reservations about indexing. Thank you for the opportunity to re-review.

Is the work clearly and accurately presented and does it cite the current literature?

Yes

If applicable, is the statistical analysis and its interpretation appropriate?

Partly

Are all the source data underlying the results available to ensure full reproducibility?

Yes

Is the study design appropriate and is the work technically sound?

Yes

Are the conclusions drawn adequately supported by the results?

Yes

Are sufficient details of methods and analysis provided to allow replication by others?

Yes

Reviewer Expertise:

NA

I confirm that I have read this submission and believe that I have an appropriate level of expertise to confirm that it is of an acceptable scientific standard.

# Peer review: Reviewer response for version 1

**DOI:** 10.5256/f1000research.57300.r100495

**Published:** 2022-01-13

**Authors:** Philipp Dahm

**Affiliations:** 1 Department of Urology, Minneapolis VAMC, University of Minnesota, Minneapolis, MN, USA

1. Abstract, Background: I don't think the first sentence makes logical sense ("although IADT was introduced...recent data suggest that those patients could be overtreated"). I think what you are trying to say is that it is being underutilized.
2. Abstract, Methods: Please clarify the denominator. Here I have the impression it is the entire (male?) population but I'd assume it to be patients with advanced/metastatic PCA. 
3. In alignment with the study results I'd say that few patients use IADT; secondarily you can state that this likely represents underuse. 
4. Introduction: I would state that metastatic disease is the main indication (not biochemical recurrence after radical prostatectomy which is also unnecessarily narrow).
5. Reference 20 seems to represent a similar study. You should discuss what this study will add/how it differs?
6. The first part of the discussion appears repetitive. If you present the results clearly, you do not need to repeat them here. Please focus on the big picture take-away points.
7. The RCTs and SRs you cite are likely overlapping bodies of evidence. Citing one recent high quality SR would suffice. Citing relevant guidelines would be even more compelling.  

Is the work clearly and accurately presented and does it cite the current literature?

Yes

If applicable, is the statistical analysis and its interpretation appropriate?

Partly

Are all the source data underlying the results available to ensure full reproducibility?

Yes

Is the study design appropriate and is the work technically sound?

Yes

Are the conclusions drawn adequately supported by the results?

Yes

Are sufficient details of methods and analysis provided to allow replication by others?

Yes

Reviewer Expertise:

evidence-based medicine, clinical practice guidelines, prostate cancer, urology

I confirm that I have read this submission and believe that I have an appropriate level of expertise to confirm that it is of an acceptable scientific standard, however I have significant reservations, as outlined above.

# Response

**Published:** 2022-04-13

**Authors:** José Antonio Cordero Rigol

Thanks Dr. Dahm for your comments and support.

 Let me answer step by step:

1. *"Abstract, Background: I don't think the first sentence makes logical sense ("although IADT was introduced...recent data suggest that those patients could be overtreated"). I think what you are trying to say is that it is being underutilized*". **Agree. Will be corrected in next version.**
2. " *Abstract, Methods: Please clarify the denominator. Here I have the impression it is the entire (male?) population but I'd assume it to be patients with advanced/metastatic PCA*." **It is correct. The prevalence estimation was referred (denominator) to the total number of patients under hormonal treatment.**
3. *"In alignment with the study results I'd say that few patients use IADT; secondarily you can state that this likely represents underuse."* **Agree. Will be corrected in next version.**
4. " *Introduction: I would state that metastatic disease is the main indication (not biochemical recurrence after radical prostatectomy which is also unnecessarily narrow).*" **Agree. Will be corrected in next version.**
5. " *Reference 20 seems to represent a similar study. You should discuss what this study will add/how it differs?*" **Agree. Basically, extension of the same method of prevalence estimation to other 3 Spanish autonomous communities confirming the same tendency previously observed in the first region (Catalonia). Will be added in next version.**
6. " *The first part of the discussion appears repetitive. If you present the results clearly, you do not need to repeat them here. Please focus on the big picture take-away points."* **Agree. The first sentence in the discussion will be removed.**
7. *"The RCTs and SRs you cite are likely overlapping bodies of evidence. Citing one recent high quality SR would suffice. Citing relevant guidelines would be even more compelling."* **Agree. The older SRs will be deleted.**

# Peer review: Reviewer response for version 1

**DOI:** 10.5256/f1000research.57300.r97868

**Published:** 2021-12-06

**Authors:** Helena Colom Codina

**Affiliations:** 1 Department of Pharmacy and Pharmaceutical technology and Physical-Chemistry, Faculty of Pharmacy and Food sciences, University of Barcelona, Barcelona, Spain

This is a very interesting study to estimate the prevalence of IAD in four Spanish autonomous communities based on electronic dispensation information from 2011 to 2016. The paper is well written; clear and appropriate in it's methods and results. A very high number of data have been recorded so that very consistent results have been obtained.

 To complete the results I would suggest to perform statistical comparisons of the prevalence values ​​between communities and between the years of follow-up. The study has fulfilled the aims, i.e. to estimate the prevalence values of IAD in different Spanish communities from 2011 to 2016; however, I would have appreciated some discussion about the reasons why the prevalence of IAD in Spain is low.

Is the work clearly and accurately presented and does it cite the current literature?

Yes

If applicable, is the statistical analysis and its interpretation appropriate?

Partly

Are all the source data underlying the results available to ensure full reproducibility?

Yes

Is the study design appropriate and is the work technically sound?

Yes

Are the conclusions drawn adequately supported by the results?

Yes

Are sufficient details of methods and analysis provided to allow replication by others?

Yes

Reviewer Expertise:

My expertise area is biopharmaceutics pharmacokynetic-pharmacodynamic data analysis and statistics

I confirm that I have read this submission and believe that I have an appropriate level of expertise to confirm that it is of an acceptable scientific standard.

# Response

**Published:** 2022-04-13

**Authors:** José Antonio Cordero Rigol

Thanks Dr. Colom for your comments and support.

 As for your suggestion " *To complete the results I would suggest to perform statistical comparisons of the prevalence values ​​between communities and between the years of follow-up.*"

 Agree. The statistical comparison will be added to the version 2 but very significant differences (p<0.001) among communities and years of follow-up will be expected due to the big size of the sample analysed. We therefore concentrated on comparing the magnitude of the differences summarised in table 2.

 With respect to your comment that " *I would have appreciated some discussion about the reasons why the prevalence of IAD in Spain is low*". Agree. This result is quite surprising and we only have conjectures to explain that e.g., Liede *at al.* reported important differences in IAD use among European countries due to "clinicians devise individualised treatment courses of optimal length based on patient characteristics while accounting for associated risks and benefits of ADT. This individualised clinical approach is represented in the variation of survey responses as it can become difficult to compartmentalise patients when deciding on treatment strategies." The fact that in Spain we have 4 physicians/1000 inhabitants may explain some extra healthcare pressure than in France, Italy or UK with >4.5 physicians/1000 inhabitants. In any case, the data are fairly consistent when it comes to demonstrating this weak use of IAD therapy in Spain.
